# Inflammatory reaction to suture materials after flexor tendon repair. A retrospective study of 594 patients

**DOI:** 10.1080/23320885.2023.2222807

**Published:** 2023-06-20

**Authors:** Marianne Arner, Linda Unge, Mikael A. Franko, Jonas Svingen

**Affiliations:** a Department of Hand Surgery, Södersjukhuset, Stockholm, Sweden; b Department of Clinical Science and Education Karolinska Institutet, Södersjukhuset, Stockholm, Sweden; c Department of Orthopedics, St Göran Hospital, Stockholm, Sweden

**Keywords:** FiberWire^®^, FiberLoop^®^, granuloma, suture material, complications, flexor tendon repair

## Abstract

We report granuloma formation after using FiberWire^®^ for flexor tendon repairs. Four subcutaneous granulomas were identified in 115 patients with FiberWire^®^ core sutures, none in 426 with braided polyester. Foreign body reactions were found in the granuloma cases. We suggest early suture removal if this specific complication is encountered.

## Introduction

During recent years, many hand surgeons have changed their preferred suture material for flexor tendon repairs, aiming for sufficient tensile strength to allow for early active motion regimes [1,2]. Braided polyester materials have been shown to be stronger and less elastic than nylon [1,3,4]. In 2011, FiberWire^®^ (Arthrex, Naples, FL, USA) was introduced on the Swedish market and *in vitro* studies have demonstrated its superior tensile strength compared to other materials [4–7]. FiberLoop^®^ is a looped suture of FiberWire^®^ consisting of a multi-strand, long chain ultra-high molecular weight polyethylene (UHMWPE) core and a braided jacket of polyester and UHMWPE.

Our hand surgery department serves an urban population of approximately two million in Stockholm, Sweden and we yearly perform around 100 flexor tendon repairs yearly in zones I and II. Before 2010, the department protocol was to use nylon sutures (Ethilon^®^, Ethicon, Johnson & Johnson, NJ, USA) for a two-strand modified Kessler repair followed by postoperative rehabilitation using a Kleinert mobilisation regime. Around 2012, the standard was changed to a four-strand braided polyester loop suture (Tsuge looped suture^®^, CrownJun, Japan) and early active mobilisation was used in most patients. Since 2016, we have gradually changed to using a four-strand FiberLoop^®^ repair, mainly due to the reported superior strength of this material, but also because we had delivery problems with the previous loop suture.

Since changing the suture material to FiberLoop^®^, we have clinically observed cases of late granuloma formation, sometimes several weeks after surgery, when skin healing has been complete. Some reports from orthopaedic practice have described similar problems with FiberWire^®^, for example in lower extremity amputations [8], Achilles tendon repairs [9], distal biceps tendon repairs [10,11] and knee cruciate ligament repairs [12]. Anecdotally, we have also heard from hand surgeons at other centres of similar cases of granuloma formation, but the incidence in flexor tendon repairs has to our knowledge not previously been reported. The aims of the present retrospective study of all flexor tendon repairs at our department performed between December 2012 and June 2019 were to investigate whether or not the change of suture material from braided polyester to FiberWire^®^ had significantly increased the incidence of granuloma formation and whether we could identify any causes for this complication.

## Patients and methods

This study is a retrospective analysis of flexor tendon repairs performed at the Department of Hand surgery at (Södersjukhuset, Stockholm, Sweden). The patient cohort in the present study was identified through the HAKIR national quality registry (www.HAKIR.se [13]). All flexor tendon repairs in zones I and II performed at our department are registered in HAKIR. In the registry, inclusion criteria are a total laceration of the flexor digitorum profundus tendon (FDP) or the flexor pollicis longus (FPL) with or without division of the superficialis (FDS). Exclusion criteria in the registry are concomitant fractures or microvascular repair. In this study we also excluded distal reinsertions and closed tendon ruptures.

The inclusion period for this study was from 03 December 2012, when registrations of flexor tendon repairs started in HAKIR, until 30 June 2019. Clinical records of all patients were thoroughly scrutinised by two of the authors (MA, LU) from April 2020 to June 2021 to allow at least two years follow-up for possible complications. Postoperative treatment for all patients included regular appointments with a physiotherapist and wound check-ups at our clinic. Flexor tendon repairs are scheduled for follow-up in HAKIR at three and 12 months postoperatively.

Variables collected from HAKIR were date of injury and date of surgery, type of injury (thumb or finger flexor tendon), injured finger and core suture number and material. Core suture materials were nylon (4-0 Ethilon^®^, Ethicon, Johnson & Johnson, NJ, USA), 3-0 or 4-0 braided polyester loop suture (Tsuge looped suture^®^, CrownJun, Japan), 4-0 FiberLoop^®^ (Arthrex, FL, USA) or in a few repairs 4-0 PDS^®^ or Prolene ^®^(Ethicon, Johnson & Johnson, NJ, USA).

Variables collected from the clinical records were smoking habits (yes, no, unknown), mechanism of injury (sharp cut or other), pre- or postoperative administration of antibiotics (yes, no, unknown), postoperative rehabilitation regime (Kleinert mobilisation, early active, immobilisation for three to four weeks or other/unknown) and the occurrence of two types of postoperative complications, as well as the results of wound cultures and pathological examinations. The two studied complications were wound healing problems (delayed skin healing or infection) and subcutaneous granuloma formation. All clinical records, including notes from nurses and therapists, were examined noting all findings not consistent with normal wound healing.

## Statistics

We chose to analyse the data based on patients and not fingers, since many of the confounding factors, such as mechanisms of injury, antibiotics and smoking habits were patient-related. In all patients with multiple injured fingers, the same suture material was used for all tendon repairs. Differences in distribution of the background variables of age group, sex, mechanism of injury, injured digit, time from injury to repair, smoking habits, preop antibiotics and rehabilitation programme between FiberLoop^®^ and Tsuge looped suture^®^, were analysed with chi-square tests. Risks of complication between FiberLoop^®^ and Tsuge looped suture were analysed with Fisher’s exact test since the number of observed complications in both groups was small.

## Results

A total of 636 patients operated with flexor tendon repairs during the study period were identified in the HAKIR registry. We excluded patients who had moved to another healthcare region and thus were lost to follow-up and two patients who had passed away shortly after surgery, in total 14 patients. We also excluded 19 closed tendon ruptures and nine patients operated with distal reinsertions (zone I). Finally, data on 594 patients operated with flexor tendon repairs in zone II in 685 fingers was analysed. The mean age for all included patients was 36.3 years (SD 15.4); median 34 (range 1–79) years and 48 (8.1%) were children below 18 years. Men constituted 69% of the patients. The mean follow-up time was 5.3 years (SD 1.9), median 5.3 (range 2.0–8.6) years.

**Figure 1.**
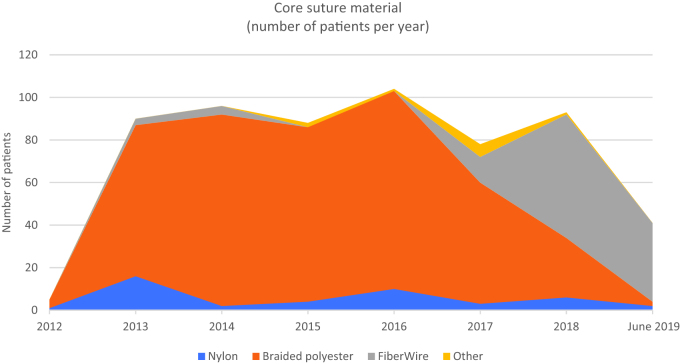
Number of patients operated per year using the different core suture materials. Observe that the inclusion period started in December 2012 and ended on 30 June 2019.

The gradual transition to using FiberLoop^®^ at our department after 2016 is visualised in Figure 1, showing the number of patients operated with the different core suture materials per year. The variables for the different groups of suture materials are listed in Table 1.

**Table 1.** Characteristics of the 594 patients operated with different suture materials for flexor tendon repair.

		FiberLoop (115)		Tsuge looped suture (426)		Other suture materials (53)		Chi-square test between FiberLoop and Tsuge looped suture
		n	%	n	%	n	%	*p* Value
Age group	<25 years	27	23.5	114	26.8	10	18.9	.50
	25–50 years	70	60.9	230	54.0	25	47.2	
	>50 years	18	15.7	76	17.8	18	34.0	
Sex	Female	34	29.6	134	31.5	18	34.0	.78
	Male	81	70.4	292	68.5	35	66.0	
Mechanism of injury	Sharp cut	110	95.7	398	93.4	48	90.6	.51
	Other	5	4.3	28	6.6	5	9.4	
Injured digit	Thumb (FPL)	20	17.4	71	16.7	10	18.9	.96
	Finger (FDP)	95	82.6	355	83.3	43	81.1	
Time from injury to repair	≤ 2 days	66	57.4	240	56.3	32	60.4	.70
	3–7 days	29	25.2	122	28.6	13	24.5	
	>7 days	20	17.4	64	15.0	8	15.1	
Smoking habits	Smoker	36	31.3	117	27.5	12	22.6	.16
	Non-smoker	96	83.5	282	66.2	40	75.5	
	Unknown	3	2.6	27	6.3	1	1.9	
Preop antibiotics	Yes	91	79.1	342	80.3	40	75.5	.95
	No	23	20.0	82	19.2	13	24.5	
	Unknown	1	0.9	1	0.2	0	0.0	
Rehab program	Early active	97	84.3	314	73.7	31	58.5	<.001
	Kleinert	1	0.9	73	17.1	12	22.6	
	Immob 3–4 weeks	15	13.0	27	6.3	9	17.0	
	Other/unknown	2	1.7	12	2.8	1	1.9	

*Note:* Total number of patients operated with the different suture materials in brackets.

In the 426 patients (501 fingers) operated with the Tsuge looped suture^®^ of braided polyester, clinical records reported on 43 cases (10.1%) with wound healing problems, of which three cases had deep infections (0.7%). In the remaining cases, skin healing was complete after extended wound care and in some instances oral antibiotics. In one patient, a longstanding pyogenic granuloma recurred in the scar after nine months and led to a reoperation. Bacterial cultures from this wound showed the growth of Enterobacter cloacae and external contamination by the patient was suspected. No cases of longstanding subcutaneous granulomas were identified in the Tsuge looped suture^®^ group.

**Figure 2.**
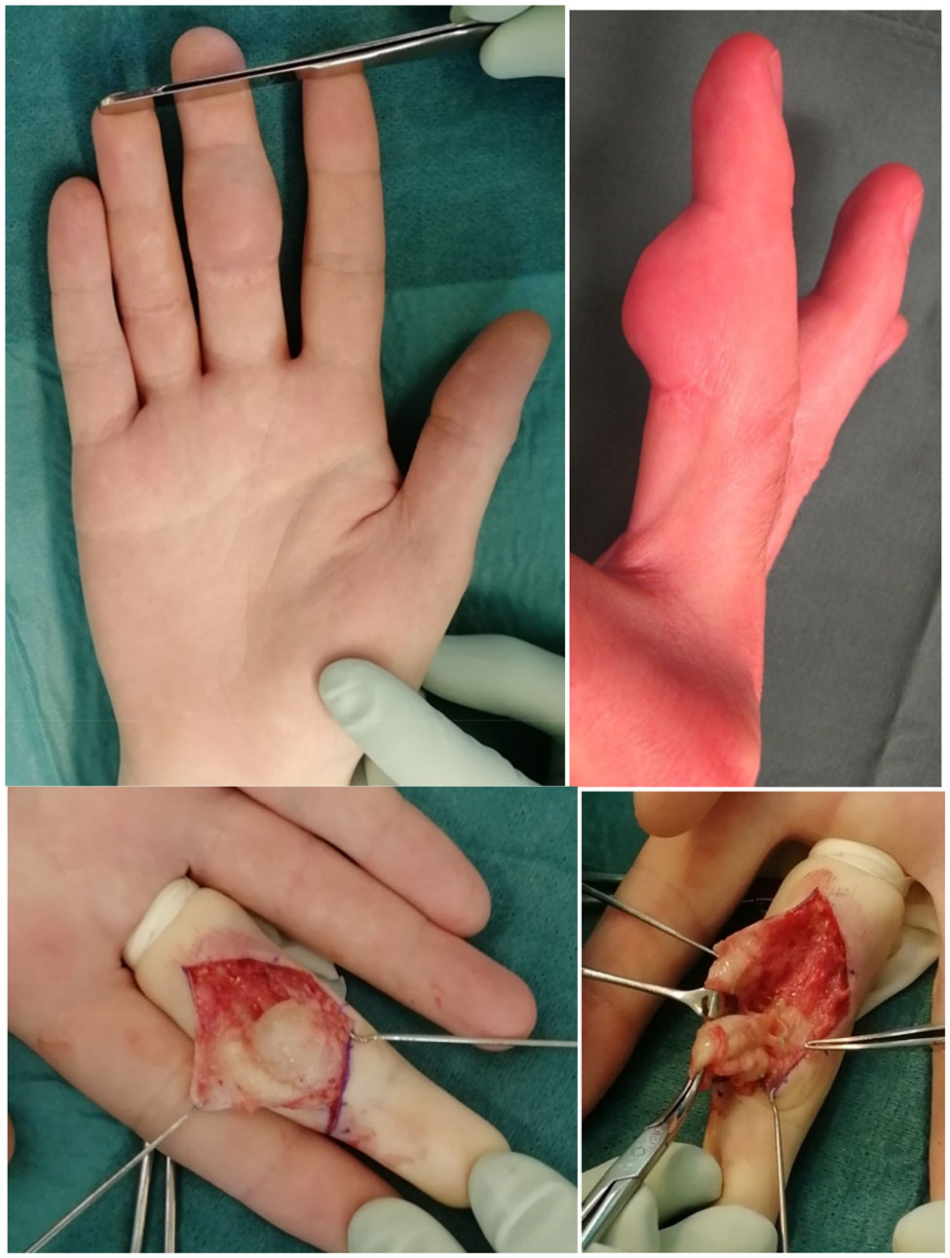
Patient number 2 in Table 2 operated with tendon repair two days after injury using 4-0 FiberLoop® sutures. After seven months, he presented with a gradually increasing volar non-tender nodule. After skin incision, a cystic granuloma was found surrounding the suture material.

**Figure 3.**
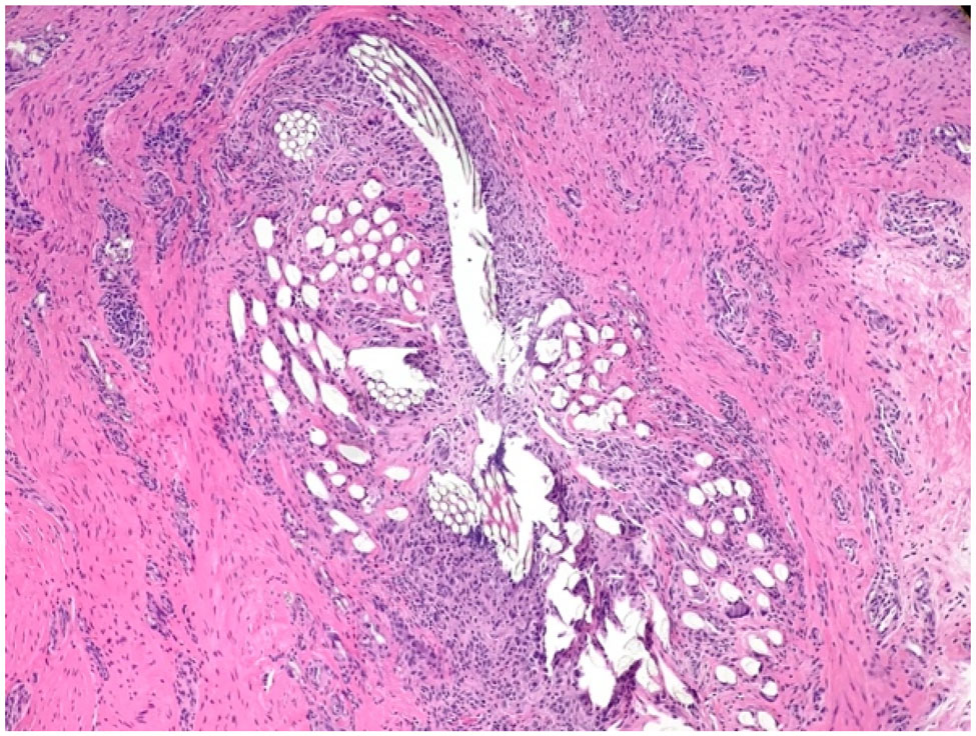
Pathological examination (patient 2) of the granulomatous material showed a pronounced inflammatory foreign body reaction. Bacterial cultures showed growth of *Staphylococcus aureus*, sensitive to isoxazolyl penicillin. Postoperatively, the patient was treated with oral flucloxacillin for one week.

In 11 out of 115 patients (123 fingers) operated with FiberLoop^®^ (9.6%), clinical records reported of wound healing problems, such as those described for the braided polyester group. One patient (0.9%) had a deep infection leading to rupture of the sutured tendon. In four patients (3.5%), longstanding subcutaneous granulomas were observed. The four patients with granulomas were reoperated at 3, 7, 9 and 30 months after the tendon repair, respectively and are presented in Table 2. One case history (patient number 2 in Table 2) is described in Figures 2 and 3. One patient had injured two fingers, but a granuloma had only occurred in one. Skin healing was complete before reoperation in three of the cases with a small exuding sinus present after the primary operation in one. Pathological examinations showed foreign body reactions in all four cases. Bacterial cultures were positive with *Staphylococcus aureus* in three of the patients. Bacterial growth was unfortunately not examined in one of the four patients. There were no tendon ruptures and healing was uneventful in three of the cases directly after removal of the FiberLoop^®^ suture. In one patient (number 4 in Table 2) who had been reoperated 30 months after the primary tendon repair, a subsequent postoperative wound infection led to another reoperation with surgical revision, but the finger then healed. After the end of the inclusion period of this study in June 2019, at least one very similar granuloma case has been diagnosed.

**Table 2.** Clinical characteristics of the four patients with granulomas.

Patient no	1	2	3	4
Age at injury (years)	44	29	26	53
Sex	Male	Male	Female	Male
Tendon injury	FPL	FDP + FDS III and FDP IV	FPL	FDP V
Mechanism of injury	Sharp cut	Compression injury	Sharp cut	Sharp cut
Time from injury to repair (days)	1	2	1	2
Smoking habits	Non smoker	Smoker	Non smoker	Non smoker
Preoperative antibiotics	Yes	Yes	Yes	No
Postoperative rehabilitation program	Early active	Early active	Immobilisation	Early active
Time from injury to reoperation (days)	214	252	71	900
Result of bacterial cultures at reoperation	No cultures taken	*Staphylococcus aureus*	*Staphylococcus aureus*	*Staphylococcus aureus*
Pathological examination	Foreign body reaction	Foreign body reaction	Foreign body reaction	Foreign body reaction
Latest clinical follow-up (months from repair)	20	12	3	80
Total active motion (TAM) at follow-up	IP + MCP = 60^o^	MCP + PIP + DIP III =235 ^o^; IV = 235 ^o^	IP + MCP = 135 ^o^	MCP + PIP + DIP =160 ^o^
Comment		Granuloma only in dig III	One year follow-up cancelled due to pandemic	Postop infection after reoperation, very stiff

The only background variable where a statistically significant difference between the FiberLoop^®^ and Tsuge looped suture^®^ groups was observed was the rehabilitation programme, but this was a result of only one patient in the FiberLoop^®^ group going through the Kleinert program. Although there were only four patients with longstanding subcutaneous granulomas, the risk of this complication was nevertheless significantly higher in the FiberLoop^®^ group with a p-value of 0.002. Since no patient presenting with the complication was found in the Tsuge looped suture^®^ group it is not possible to obtain an estimate of the odds ratio, but the lower limit in a 95% confidence interval was 2.5 indicating at least a more than doubled risk.

## Discussion

This is a retrospective study of a large consecutive cohort of patients operated with flexor tendon repair, with a focus on granuloma formation related to the core suture material. Wound healing problems were not uncommon, but in the majority of cases they were managed by conventional wound care procedures. The rate of deep infections was low in both groups. The type of foreign body reaction and subcutaneous granuloma formation related to FiberLoop^®^ sutures was rare and of a different nature than previously observed for other core suture materials. This complication usually presented late, after skin healing, as a non-tender lump in the operated area. At reoperation, positive bacterial cultures were found in most cases, but usually healing was uneventful after removal of the suture, and infection did not appear to be the main cause of the granuloma. In an experimental study on mouse skin, the bacterial concentration of tissues surrounding braided sutures was significantly greater than encountered with monofilament sutures [14]. Colonisation with strains of *S. aureus* of low virulence might have contributed to the late granuloma formation in our patient cohort. However, since both the Tsuge looped suture^®^ and the FiberLoop^®^ sutures are braided, this does not explain the differences in the rate of granuloma formation. In our study, the extent of use of pre- or postoperative antibiotics was not statistically different between the two cohorts operated with Tsuge looped ^®^ or FiberLoop^®^ sutures.

We did not find any equivalent case of granuloma formation among the 426 patients operated with the Tsuge looped suture^®^ and none among the few patients where nylon sutures had been used. Granuloma formation thus seems to be a different type of complication, not observed in flexor tendon repairs before the introduction of FiberLoop^®^. Yokoe et al. [12] recently reported that atopic dermatitis might be a contributing factor to the inflammatory response to FiberWire^®^ in cruciate ligament repairs; however, none of our patients had this condition. Ollivere et al. [9] advised against the use of FiberWire^®^ for Achilles tendon repairs after encountering one case of granuloma formation eight months after the primary surgery. They suggested that both the silicone coating and the polyethylene core might be responsible for the foreign body reaction. The issue of the silicone coating of FiberWire^®^ was also mentioned by Mack et al. [8] reporting granuloma formation after lower extremity amputations. In a rabbit study, it was reported that Ethibond^®^ caused the most severe inflammatory reaction in tendon and muscle at three weeks, but the lowest at six weeks [15]. FiberWire^®^ in that study caused the least severe reaction at three weeks, but more than Ethibond^®^ at six weeks. An *in vitro* study using cell cultures, examined the induction of gene expression and inflammatory mediator release by different suture materials [16]. Vicryl^®^, Ti-Cron^®^ and FiberWire^®^ caused upregulation of proinflammatory markers genes early, while Monocryl^®^, Ethilon ^®^and Ethibond Excel ^®^remained reasonably inert.

There is at present no clear consensus on the best choice of suture material for flexor tendon repairs and choices usually seem to be guided by surgeons’ personal preferences [2]. Factors such as tensile strength, elasticity and knot-holding characteristics are all important, but tissue friendliness may be another factor to be considered. The statistical analysis of data in the present study indicated at least a doubled risk of developing a granuloma when using FiberLoop^®^ sutures and we could not identify any obvious confounding factors for this complication. However, since we observed only four cases with granuloma in the FiberLoop^®^ group and none in the braided polyester group we could not statistically assess the influence of potential confounders.

A limitation of the present study is the retrospective design and the fact that other changes in clinical routines may have taken place during the long inclusion period of 7.5 years. Another factor is that clinical records have flaws, sometimes making it difficult to judge the occurrence and severity of complications. We have tried to be meticulous and to interpret the records as objectively as possible.

A strength of the study is that all patients with flexor tendon injuries were followed at least up to three months postoperatively in our department. Patients were also offered a one-year follow-up through the HAKIR registry. The pandemic starting in 2020 unfortunately made these follow-ups less frequent, however patients experiencing a complication would most probably be cared for at the department.

In conclusion, we have observed a rare, but specific complication that is most probably related to the use of FiberLoop^®^ as the core suture for flexor tendon repairs. This infrequent problem must be weighed against the advantage of using a very strong suture material. However, we encourage hand surgeons to be aware of the complication and we recommend early removal of the suture material when encountering granuloma formation after tendon repairs using FiberLoop^®^. More studies are needed to elucidate possible contributing factors, such as atopic dermatitis, delay to surgery causing bacterial colonisation, more active rehabilitation regimes or variation in surgical techniques.
